# Cutaneous lesions in the setting of hypophosphatasia

**DOI:** 10.1016/j.jdcr.2023.10.001

**Published:** 2023-10-11

**Authors:** Nancy W. Shen, Lauren G. Yi, Wilson Omesiete, Christina M. Peroutka, Shyam S. Raghavan, Kenneth E. Greer

**Affiliations:** a University of Virginia School of Medicine, Charlottesville, Virginia; b University of Virginia Department of Dermatology, Charlottesville, Virginia; c University of Virginia Department of Pediatrics, Charlottesville, Virginia; d University of Virginia Department of Pathology, Charlottesville, Virginia

**Keywords:** asfotase alfa, calcinosis cutis, genetic disorders, neonatal, pediatric dermatology

## Introduction

Hypophosphatasia (HPP) is an ultrarare genetic condition caused by pathogenic variants in the gene for tissue-nonspecific alkaline phosphatase (ALPL), resulting in age- and sex-specific low serum alkaline phosphatase (ALP) activity levels.^1^^,^^2^ Historically, HPP is classified into 6 forms based on the age of onset and severity of presentation, and the most severe forms of HPP have an estimated disease prevalence of 1 in 100,000 to 1 in 400,000, whereas population allele frequencies suggest a much higher prevalence of 1 in 6370 for all forms.^2^, ^3^, ^4^ Common clinical manifestations in adult patients include osteomalacia and early loss of teeth.^2^^,^^3^^,^^5^ However, because disease onset and symptoms are highly variable, the diagnosis of HPP is challenging.

## Case report

**Fig 1:**
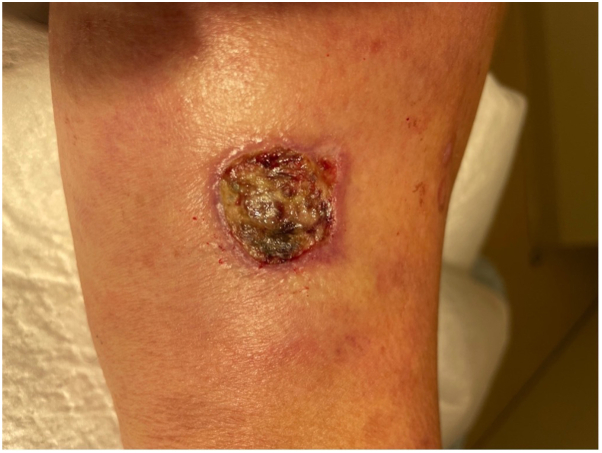
Appearance of painful ulcer at first presentation to clinic.

A 61-year-old White woman with a history of childhood-onset HPP presented to dermatology clinic with a 1-month history of a nonhealing, painful ulcer on the upper portion of the arm. The lesion started as a painful subcutaneous nodule that progressed to ulceration over the course of many weeks. Physical examination was notable for a 2-cm tender ulcer with raised, violaceous border on the right triceps in addition to multiple firm subcutaneous nodules on the bilateral elbows (Fig 1).

**Fig 2:**
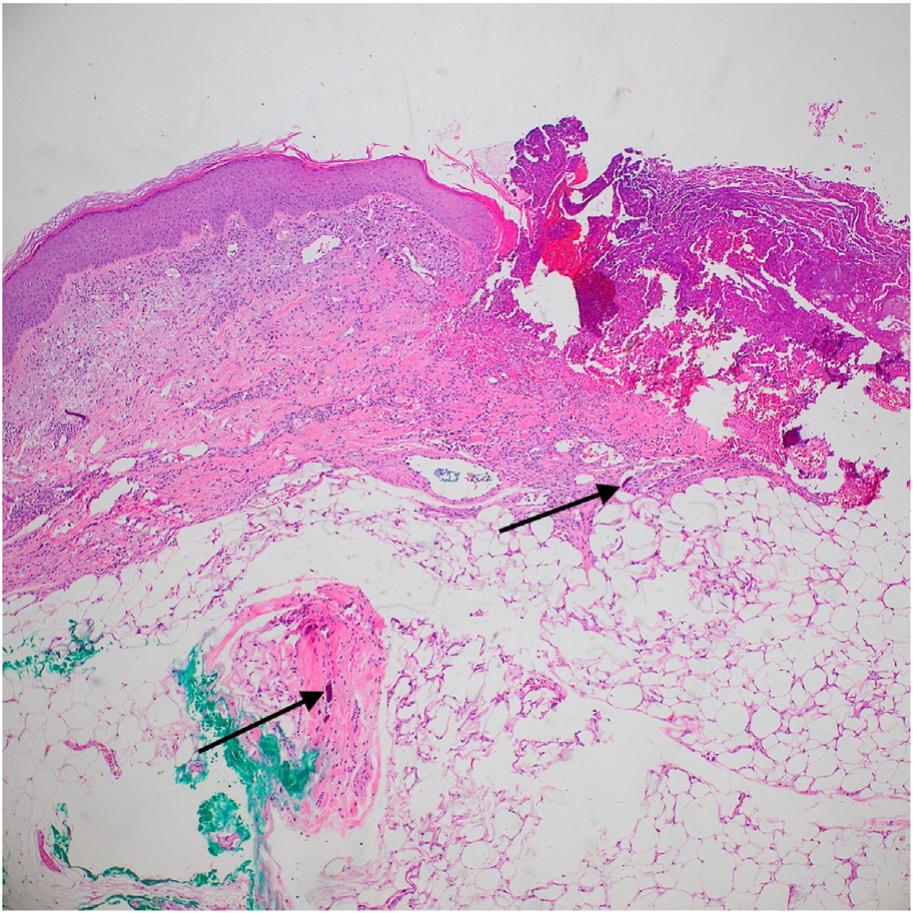
Hematoxylin-eosin stain (Original magnification: ×4). Arrows indicating calcium deposition in the dermis and subcutaneous tissue.

**Fig 3:**
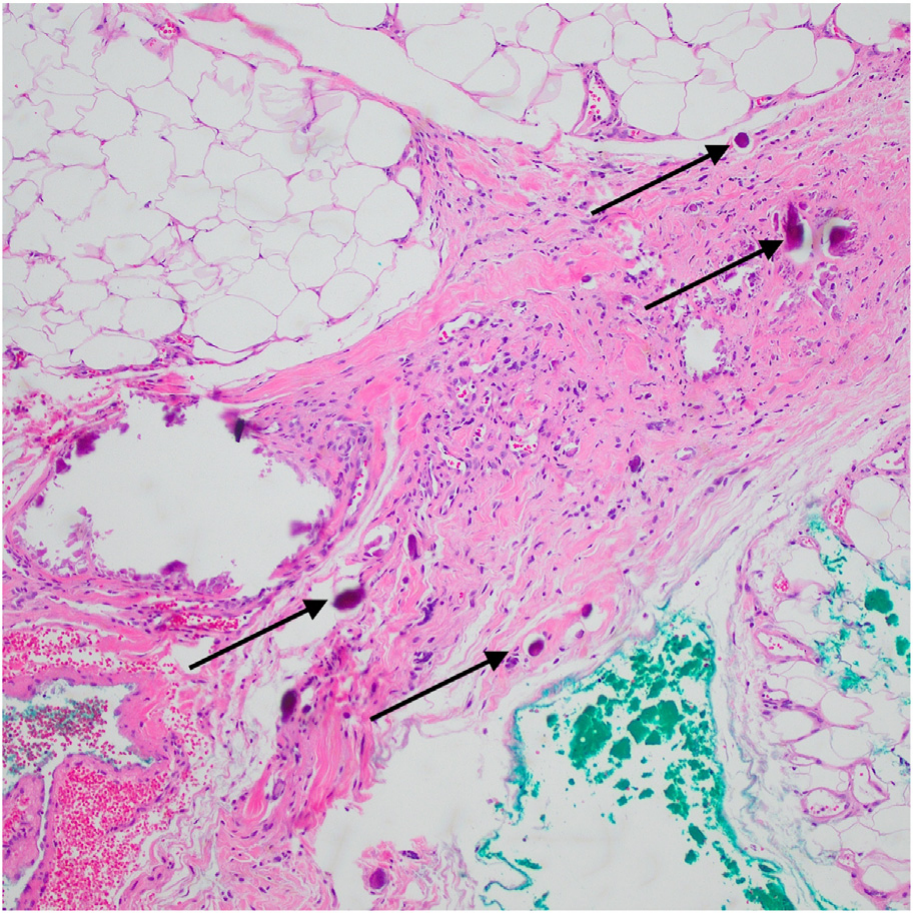
Hematoxylin-eosin stain (Original magnification: ×10). Arrows indicating abundant calcification within the dermal collagen, vessels, and subcutaneous fat.

**Fig 4:**
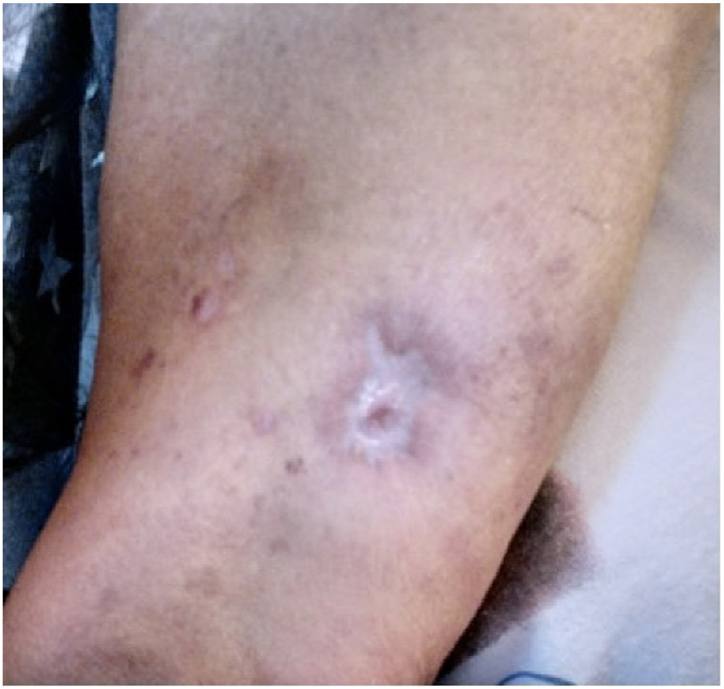
Healed ulcer on the right triceps after treatment with topical silvadene.

An incisional biopsy was performed at the edge of the ulcer, and histopathology revealed mixed inflammation and abundant calcium deposition within the dermal collagen and subcutaneous fat (Figs 2 and 3). Periodic acid–Schiff staining was negative for fungal elements. There was no growth on bacterial, mycobacterial, or fungal tissue cultures. The ulcer was treated with topical silver sulfadiazine, which resulted in healing (Fig 4).

Although the patient was not formally diagnosed with HPP until adulthood, she had a long history of weakness, chronic migraines, bowed bones, poor growth, and short stature that was most consistent with childhood-onset HPP. She lost all teeth by 21 years of age, and her adult musculoskeletal complications included vertebral compression fractures and ligamentous calcification in the spine. She denied a history of previous skin disorders. The patient’s son, niece, and grandson also had a confirmed diagnosis of HPP. Genetic testing in the patient revealed a likely pathogenic variant in ALPL c.1427A>G (p.Glu476Gly) in the setting of a persistently low serum ALP levels. After her diagnosis at the age of 60 years, the patient was started on asfotase alfa (AA), a synthetic human ALP that is Food and Drug Association–approved to treat patients with perinatal and infantile- and juvenile-onset HPP.^3^

Notably, our patient was receiving AA injections for 2 years before the current presentation at a dose of 1 mg/kg 6 days per week, with onset of subcutaneous nodules in her arms after starting these injections. Interestingly, the patient has continued to develop new nodules without ulceration in her arms despite only administering injections in her abdomen and occasionally in her thighs, where nodules have not developed.

## Discussion

HPP is an ultrarare metabolic bone disorder that may be unfamiliar to many dermatologists. It is caused by disease-causing mutations in the gene encoding the tissue-nonspecific isoenzyme of ALPL with reduction in ALP activity levels. Decreased ALP activity allows excess inorganic pyrophosphate (PPi) to accumulate in the extracellular matrix, resulting in primarily bone and dental disease.^6^

There are a few considerations for the cause of the patient’s ulcer. Given that she began developing subcutaneous nodules after starting AA, her cutaneous manifestations may be a reaction to the medication. Alternatively, given her history of ligamentous calcification in her spine before treatment with AA, the findings of calcium deposition within our patient’s soft tissues may also be due to her underlying HPP and its associated alterations in calcium and phosphorus metabolism with secondary foreign body reaction and ulceration. Other diagnoses considered at initial presentation included pyoderma gangrenosum (PG) and calciphylaxis. The presence of additional subcutaneous nodules decreased clinical suspicion of PG. Calciphylaxis was also less likely given that the patient did not have any associated conditions, such as hyperparathyroidism or chronic kidney disease. Furthermore, calcification was present both inside and outside the vasculature.

Given the rarity of this disease, the available data regarding skin findings in patients with HPP are minimal. Individuals with HPP are at an increased risk of developing ectopic calcifications, especially of the cornea and kidneys (nephrocalcinosis and nephrolithiasis), and it is unknown whether treatment with AA exacerbates or ameliorates this risk.^7^ Of the 30 individuals followed-up at our institution, this patient is the only one with cutaneous manifestations of the disorder.

We conducted a review of the literature and found very few studies describing the cutaneous manifestations of this disease. In terms of clinical symptoms, an article reviewing neonatal and pediatric cases of HPP noted characteristic skin dimpling in neonates.^8^ This finding occurs due to tethering of soft tissue at sites of bone spurs, usually in the forearms and legs, because all patients with HPP documented in the literature with skin dimpling also had bowed bones.^8^^,^^9^ Importantly, there are 2 documented cases of calcinosis cutis after AA therapy. Charalambides et al^10^ reported a case of severe injection site reaction to subcutaneous injections of AA in a patient with juvenile-onset HPP receiving 60 mg AA for 6 days per week for 6 months alternating in the abdomen and thigh.^10^ Skin biopsies at these injection sites showed calcium deposition in the dermis, subcutis, vessel walls, and sweat ducts with a multinucleate giant cell response. The pathophysiology of this reaction is due to degradation of PPi by synthetic ALP found in AA; degraded PPi produces higher levels of inorganic phosphate, which promote calcification.^10^

In conclusion, HPP is a heritable, metabolic disorder with pleiotropic manifestations most commonly affecting bone and tooth mineralization. It has rare cutaneous manifestations, with the most prominent reported feature being skin dimpling, particularly in the infantile and juvenile forms of the disease. Treatment with AA enzyme therapy is the only definitive treatment for HPP. Although ectopic calcifications of the eye or kidney are known complications of HPP, their relatedness with therapy is unknown. Injection site reactions of pain, pruritus, erythema, and lipoatrophy are described in patients treated with AA, with 2 rare cases of calcinosis cutis published. To our knowledge, there are no other reports of cutaneous manifestations of HPP or reports of PG or distant dermal ectopic calcification related to therapy published in the literature.

## Conflicts of interest

None disclosed.
